# Diagnostically Challenging Disseminated Bacillus Calmette–Guerin Infection With Lung and Liver Involvement: A Case Report

**DOI:** 10.1002/rcr2.70448

**Published:** 2025-12-18

**Authors:** Shinichi Chang, Makiko Yomota, Ayumi Takizawa, Noriyo Yanagawa, Tsunekazu Hishima, Naoko Kubota, Yukio Hosomi

**Affiliations:** ^1^ Department of Respiratory Medicine Tokyo Metropolitan Cancer and Infectious Diseases Center, Komagome Hospital Tokyo Japan; ^2^ Department of General Medicine Tokyo Metropolitan Cancer and Infectious Diseases Center, Komagome Hospital Tokyo Japan; ^3^ Department of Radiology Tokyo Metropolitan Cancer and Infectious Diseases Center, Komagome Hospital Tokyo Japan; ^4^ Department of Pathology Tokyo Metropolitan Cancer and Infectious Diseases Center, Komagome Hospital Tokyo Japan

**Keywords:** Bacillus Calmette–Guerin, diffuse micronodules, disseminated BCG infection, intravesical BCG therapy, noncaseating granulomas

## Abstract

Intravesical Bacillus Calmette–Guerin (BCG) therapy is the standard treatment for non–muscle‐invasive bladder cancer. While it is effective, it sometimes causes a disseminated BCG infection, which is often difficult to diagnose microbiologically. We report herein a 73‐year‐old, male patient in whom a persistent fever, dyspnoea, and jaundice developed after he received his tenth round of intravesical BCG therapy. Chest computed tomography revealed diffuse micronodules, and lung and liver biopsies demonstrated multiple, well‐formed, non‐caseating granulomas. Despite negative microbiological test results, a disseminated BCG infection was suspected. Antimycobacterial therapy was started but discontinued after 2 weeks due to a drug‐induced eruption. The clinical and radiological findings continued to improve without further treatment. The present case highlights the importance of a detailed assessment of radiological and histopathological findings for the diagnosis and management of diffuse lung diseases, including complications related to intravesical BCG therapy.

## Graphical abstract

Disseminated Bacillus Calmette–Guerin (BCG) infection is a rare but serious complication of intravesical BCG therapy and is often difficult to diagnose due to the low sensitivity of microbiological tests. In the present case, a qualitative assessment of radiological and histopathological findings enabled the diagnosis.

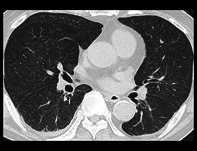

## 1 Introduction

Intravesical Bacillus Calmette–Guerin (BCG) instillation is effective for preventing superficial bladder cancer recurrences. However, in rare instances, it can result in a disseminated BCG infection. Microbiological tests often return negative, making a definitive diagnosis challenging and complicating treatment decisions. Herein we report a diagnostically challenging case of suspected disseminated BCG infection which was finally diagnosed on the basis of a qualitative assessment of radiological and histopathological findings.

## 2 Case Report

A 73‐year‐old, male patient with a history of hypertension underwent a transurethral resection of a T1 high‐grade bladder tumour, after which he received intravesical BCG therapy. Two years later, after receiving his tenth round of the treatment, he presented with a fever, for which he received a 3‐day course of oral levofloxacin. However, his fever persisted for 3 weeks, during which dyspnoea and jaundice developed, prompting a referral to our hospital's Department of General Medicine. On presentation, the patient had body temperature 36.5°C, blood pressure 88/58 mmHg, heart rate 110 beats/min, and peripheral oxygen saturation 90% on room air. A physical examination revealed scleral icterus. Laboratory tests demonstrated elevated hepatobiliary enzymes, predominantly alkaline phosphatase, and total bilirubin. The serum immunoglobulin values were normal, but KL‐6 and SP‐A were mildly elevated (Table 1). Antinuclear antibody, anti‐mitochondrial antibody, beta‐D‐glucan, and T‐SPOT were negative. He had no history of tuberculosis.

**TABLE 1:** Laboratory data of this case.

Laboratory data	Value	Reference range	Unit
Haematology			
White blood cell	3500	3300–8600	/μL
Haemoglobin	15.1	13.7–16.8	g/dL
Platelets	17.8	15.8–34.8	10^4^/μL
Serology			
Total bilirubin	3.0	0.4–1.5	mg/dL
AST	75	13–30	U/L
ALT	65	10–42	U/L
ALP	481	39–113	U/L
CRP	2.4	< 0.14	mg/dL
KL‐6	1100	< 500	U/mL
SP‐A	94.3	< 43.8	ng/mL
Immunoglobulin G	1867	861–1747	mg/mL
Immunoglobulin A	334	93–393	mg/mL
Immunoglobulin M	114	33–83	mg/mL
Antinuclear antibody	Negative		
Anti‐mitochondrial antibody	Negative		
Beta‐D‐glucan	Negative		
T‐SPOT	Negative		

Abbreviations: ALP, alkaline phosphatase; ALT, alanine aminotransferase; AST, aspartate aminotransferase; CRP, C‐reactive protein; KL‐6, Krebs von den Lungen‐6; SP‐A, surfactant protein‐A.

**FIGURE 1:**
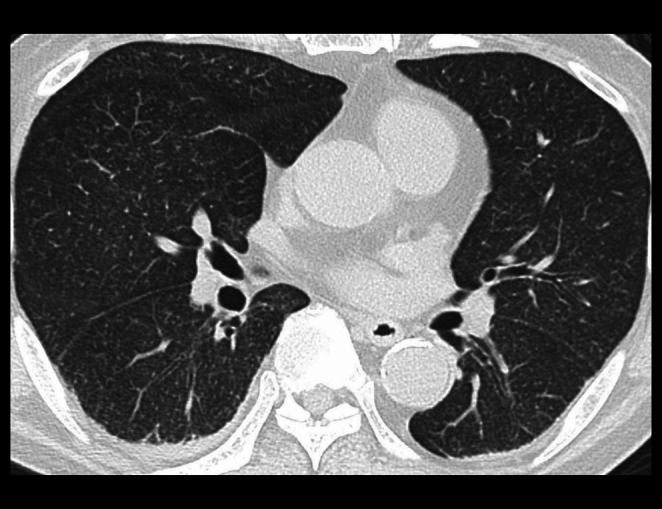
Chest computed tomography (slice thickness: 1 mm) revealed micronodules with a diffuse distribution unrelated to the lobular structure, uniformly distributed throughout the lungs.

Although a chest X‐ray found no obvious abnormalities, chest computed tomography (CT) revealed micronodules with a diffuse distribution unrelated to the lobular structure and uniformly distributed throughout the lungs (Figure 1). Contrast‐enhanced abdominal CT demonstrated hepatomegaly and a periportal low attenuation.

**FIGURE 2:**
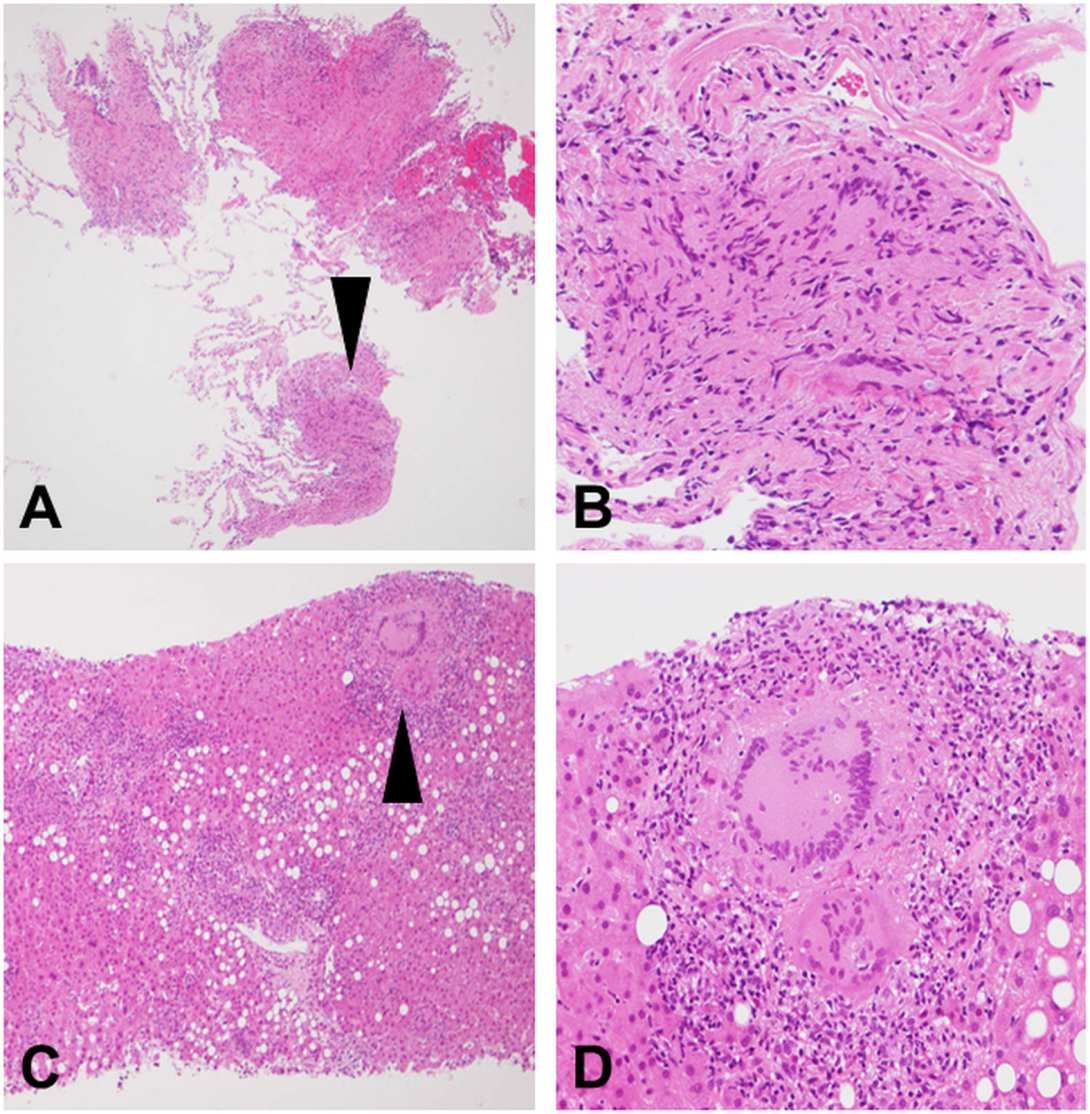
Transbronchial lung biopsy revealed epithelioid granulomas diffusely distributed in the bronchiolar walls, perivenular interstitium, and alveolar spaces (haematoxylin and eosin staining, low power) (A). The epithelioid granulomas (arrowhead) were well‐formed and non‐caseating. No necrosis was found (haematoxylin and eosin staining, high power) (B). A liver biopsy also found scattered epithelioid granulomas distributed not only in the lobular area but also in the portal area (haematoxylin and eosin staining, low power) (C). The epithelioid granulomas (arrowhead) with Langhans giant cells were accompanied by lymphocytic infiltration (haematoxylin and eosin staining, high power) (D).

As Ziehl–Neelsen staining and a polymerase chain reaction (PCR) test of a sputum sample were negative, the differential diagnosis of diffuse lung diseases was unable to be established based on the CT findings alone. Therefore, bronchoscopy and a liver biopsy were performed. However, the Ziehl–Neelsen staining, PCR test, and mycobacterial cultures of blood, sputum, and biopsy specimens from the lung and liver returned negative. A transbronchial lung biopsy demonstrated findings typical of a mycobacterial infection, consisting of multiple, diffusely distributed, well‐formed, non‐caseating epithelioid granulomas without any evidence of necrosis (Figure 2). The liver biopsy specimens also demonstrated similar granulomas accompanied by Langhans giant cells (Figure 2).

As the patient's respiratory status and liver function gradually improved despite the lack of a definitive diagnosis 2 months after the intravesical BCG therapy, the patient was managed conservatively without treatment. Although all the microbiological tests were negative, disseminated BCG infection was considered the most likely diagnosis based on the clinical presentation and radiological and pathological findings. Antimycobacterial treatment with isoniazid 300 mg, rifampicin 600 mg, and ethambutol 1000 mg was begun. On day 14 the antimycobacterial treatment was discontinued after a generalised, drug‐induced eruption developed. However, the patient's condition has remained stable without further treatment.

## 3 Discussion

Intravesical BCG therapy is strongly recommended as a treatment for newly diagnosed carcinoma in situ, high‐grade T1 and high‐risk Ta bladder cancer [1]. However, intravesical BCG therapy is associated with rare but serious, systemic adverse events, including pulmonary complications, such as disseminated BCG infection and hypersensitivity pneumonitis [2]. Diagnosing a disseminated BCG infection is challenging owing to the poor sensitivity of microbiological tests, the positivity rate being 25.3% for acid‐fast bacilli staining, 40.9% for mycobacterial cultures, and 41.8% for PCR‐based assays [2]. When microbiological confirmation cannot be achieved, the diagnosis often relies on the detection of pulmonary micronodules on CT and of epithelioid granulomas in a biopsy specimen [2]. However, these findings are non‐specific and do not reliably distinguish disseminated BCG infection from other, diffuse lung diseases, such as tuberculosis, sarcoidosis and hypersensitivity pneumonitis. In the present case, detailed, qualitative assessment of radiological and pathological findings helped exclude these diseases and led to the presumptive diagnosis of disseminated BCG infection. Tuberculosis was considered unlikely due to the consistently negative microbiological results.

Micronodules occur on CT images of a variety of diseases. However, differences in their pattern of distribution serve as important clues for the differential diagnosis. In hematogenous infections, micronodules typically have a diffuse, uniform distribution without perilymphatic, lobar, or regional predominace [3]. In contrast, sarcoidosis typically presents with micronodules with a perilymphatic pattern of distribution [3]. Hypersensitivity pneumonitis usually presents with poorly defined, centrilobular micronodules but in some cases may also be diffusely distributed [3]. In the present case, CT demonstrated well‐defined micronodules with a diffuse distribution throughout all the lobes, thus raising the index of suspicion for a disseminated BCG infection. Because micronodules are a non‐specific finding, detailed assessment of their distribution pattern is essential to the differential diagnosis.

Furthermore, histopathological findings can contribute significantly to the diagnostic process. An infection should always be suspected whenever necrosis is found within granulomas; however, the absence of necrosis and negative staining results do not necessarily rule out an infection [4]. Qualitative features of granulomas also facilitate the differential diagnosis; granulomas associated with mycobacterial infections and sarcoidosis are typically well‐formed whereas those associated with hypersensitivity pneumonitis are generally small and poorly cohesive [4]. In the present case, a histological examination revealed well‐formed, non‐caseating granulomas, which alone were insufficient to distinguish reliably between disseminated BCG infection and sarcoidosis. However, biopsy samples were obtained from seven sites across the upper, middle, and lower lobes. This broad sampling of tissue revealed diffusely distributed granulomas consistent with a disseminated BCG infection.

In conclusion, a comprehensive assessment integrating radiological and histopathological findings is important for the diagnosis and management of diffuse lung diseases, including complications of intravesical BCG therapy.

## Author Contributions

Shinichi Chang wrote the first draft. Makiko Yomota, Ayumi Takizawa, Noriyo Yanagawa, Tsunekazu Hishima, Naoko Kubota, and Yukio Hosomi revised the article for important intellectual content. All the authors have approved the final version.

## Consent

The authors declare that written informed consent was obtained for the publication of this manuscript and accompanying images and attest that the form used to obtain consent from the patient complies with the journal's requirements as outlined in the author guidelines.

## Conflicts of Interest

Dr. Makiko Yomota received honoraria (lecture fees) from AstraZeneca, Takeda, MSD, Chugai Pharmaceutical, Ono Pharmaceutical, and Bristol‐Myers Squibb outside the submitted work. Dr. Yukio Hosomi received honoraria (lecture fees) from AstraZeneca, Eli Lilly Japan, Taiho Pharmaceutical, Chugai Pharmaceutical, Ono Pharceutical, Bristol‐Myers Squibb, Kyowa Kirin, Nippon Kayaku, Takeda, Eisai, Novartis, Pfizer, and MSD outside the submitted work. The other authors have nothing to disclose.

## Data Availability

Data sharing not applicable to this article as no datasets were generated or analysed during the current study.
